# Supplementary material for: Serious adverse reactions associated with ivermectin: A systematic pharmacovigilance study in sub-Saharan Africa and in the rest of the World
Source: PLoS Negl Trop Dis. 2021 Apr 20;15(4):e0009354. doi: 10.1371/journal.pntd.0009354 (PMC8087035; doi:10.1371/journal.pntd.0009354)
Supplement: S1 Table — A. Most frequently reported serious ADRs for each System Organ Class (SOC). If several sADRs belonging to the same SOC are reported in a single patient (ICSR form), the SOC is counted only once in the total. B. Most frequently reported serious ADRs for each System Organ Class (SOC) in SSA. If several sADRs belonging to the same SOC are reported in a single patient (ICSR form), the SOC is counted only once in the total. C. Most frequently reported serious ADRs for each System Organ Class (SOC) in RoW. If several sADRs belonging to the same SOC are reported in a single patient (ICSR form), the SOC is counted only once in the total. (DOCX) [file pntd.0009354.s001.docx]

**S1A Table. Most frequently reported serious ADR associated with ivermectin for each System Organ Class (SOC).** If several sADRs belonging to the same SOC are reported in a single patient (ICSR form), the SOC is counted only once in the total.

| **SOC (number of serious ADR reports)** | **Most frequently reported serious ADR** | | | | |
| --- | --- | --- | --- | --- | --- |
|  | 1^st^ reported (n) | 2^nd^ reported (n) | 3^rd^ reported (n) | 4^th^ reported (n) | 5^th^ reported (n) |
| **General disorders and administration site conditions (296)** | Asthenia (78) | Pyrexia (63) | Drug ineffective (38) | Gait disturbance (28) | Pain (19) |
| **Nervous system disorders (209)** | Headache (82) | Dizziness (43) | Coma (36) | Depressed level of consciousness (20) | Loss of consciousness (16) |
| **Skin and subcutaneous tissue disorders (203)** | Pruritus (76) | Rash (31) | Erythema (15) | Stevens-Johnson syndrome (15) | Rash maculo-papular (14) |
| **Gastrointestinal disorders (129)** | Vomiting (34) | Diarrhea (30) | Abdominal pain (23) | Nausea (12) | Dysphagia (9) |
| **Infections and infestations (82)** | Strongyloidiasis (34) | Pneumonia (11) | Sepsis (7) | Conjunctivitis (5) | Acarodermatitis (5) |
| **Musculoskeletal, connectives tissues disorders (78)** | Back pain (28) | Arthralgia (27) | Myalgia (20) | Pain in extremity (10) | Musculoskeletal pain (4) |
| **Injury, poisoning, procedural complications (67)** | Off label use (15) | Accidental exposure (10) | Incorrect route of administration (6) | Exposure during pregnancy (4) | Product use in unapproved indication (4) |
| **Psychiatric disorders (62)** | Confusional state (13) | Abnormal behavior (11) | Agitation (9) | Disorientation (5) | Hallucination (4) |
| **Respiratory, thoracic, mediastinal disorders (62)** | Dyspnea (18) | ARDS (7) | Cough (7) | Respiratory failure (5) | Asthma (4) |
| **Renal and urinal disorders (57)** | Urinary incontinence (21) | Acute kidney injury (8) | Chromaturia (4) | Oliguria (4) | Hematuria (3) |
| **Investigations (57)** | Weight decreased (5) | Hepatic enzyme increased (5) | Platelet count decreased (4) | CRP increased (3) | Heart rate increased (3) |
| **Eye disorders (49)** | Conjunctival haemorrhage (21) | Eye pain (5) | Vision blurred (4) | Visual impairment (4) | Blindness (3) |
| **Hepatobiliary disorders (48)** | Hepatitis (10) | Hepatocellular injury (9) | Jaundice (5) | Liver disorder (5) | Hepatic failure (4) |
| **Vascular disorders (47)** | Hypotension (20) | Hypertension (7) | Hematoma (3) | Circulatory collapse (2) | Orthostatic hypotension (2) |
| **Blood and lymphatic system disorders (44)** | Eosinophilia (18) | Lymphadenopathy (5) | Agranulocytosis (4) | Anemia (4) | Thrombocytopenia (4) |
| **Cardiac disorders (28)** | Cardiac arrest (5) | Palpitations (5) | Tachycardia (5) | Cardiac failure (4) | Cardio-respiratory arrest (3) |
| **Metabolism and nutrition disorders (24)** | Decreased appetite (5) | Hyperkaliemia (4) | Dehydration (3) | Hyponatremia (3) | Hyperglycemia (2) |
| **Immune system disorders (15)** | Hypersensitivity (7) | Anaphylactic shock (4) | Anaphylactic reaction (1) | Drug hypersensitivity (1) | Immune system disorder (1) |
| **Ear and labyrinth disorders (14)** | Vertigo (9) | Ear pain (2) | Tinnitus (2) | Deafness (1) | Ototoxicity (1) |
| **Reproductive system and breast disorders (9)** | Pelvic pain (2) | Scrotal oedema (2) | Menstruation irregular (1) | Oedema genital (1) | Penile swelling (1) |
| **Pregnancy, puerperium, perinatal disorders (9)** | Abortion spontaneous (4) | Foetal death (2) | Oligohydramnios (1) | Premature delivery (1) | Stillbirth (1) |
| **Neoplasm benign, malignant, unspecified (8)** | Hodgkin’s disease (3) | Hydatidiform mole (1) | Large B-cell lymphoma (1) | Glioblastoma (1) | T-cell lymphoma (1) |
| **Endocrine disorders (7)** | Hypothyroidism (2) | Adrenal insufficiency (1) | Diabetes insipidus (1) | Adrenocortical insufficiency (1) | Autoimmune thyroiditis (1) |
| **Social circumstances (4)** | Loss of independence (2) | Immobile (1) | Impaired driving ability (1) |  |  |
| **Surgical and medical procedures (4)** | Central catheterization (1) | Limb operation (1) | Spinal operation (1) | Mechanical ventilation (1) | Endotracheal intubation (1) |
| **Product issues (3)** | Product taste abnormal (1) | Wrong label (1) | Product substitution issue (1) | Product availability issue (1) |  |
| **Congenital, familial and genetic disorders (1)** | Congenital anomaly (1) |  |  |  |  |

**S1B Table. Most frequently reported serious ADR associated with ivermectin for each System Organ Class (SOC) in SSA.** If several sADRs belonging to the same SOC are reported in a single patient (ICSR form), the SOC is counted only once in the total.

| **SOC (number of serious ADR reports)** | **Most frequently reported serious ADR** | | | | |
| --- | --- | --- | --- | --- | --- |
|  | 1^st^ reported (n) | 2^nd^ reported (n) | 3^rd^ reported (n) | 4^th^ reported (n) | 5^th^ reported (n) |
| **General disorders and administration site conditions (120)** | Asthenia (61) | Pyrexia (42) | Gait disturbance (22) | Pain (15) | Chills (9) |
| **Nervous system disorders (112)** | Headache (60) | Coma (31) | Dizziness (29) | Depressed level of consciousness (13) | Loss of consciousness (7) |
| **Skin and subcutaneous tissue disorders (72)** | Pruritus (47) | Rash (9) | Steven-Johnson syndrome (8) | Rash pruritic (4) | Angioedema (2) |
| **Musculoskeletal, connectives tissues disorders (52)** | Back pain (22) | Arthralgia (20) | Myalgia (14) | Musculoskeletal pain (3) | Neck pain (3) |
| **Gastrointestinal disorders (51)** | Diarrhea (21) | Vomiting (17) | Abdominal pain (9) | Anal incontinence (5) | Abdominal distension (2) |
| **Eye disorders (28)** | Conjunctival haemorrhage (19) | Eye pain (5) | Corneal opacity (1) | Eye discharge (1) | Eye swelling (1) |
| **Renal and urinal disorders (28)** | Urinary incontinence (19) | Oliguria (3) | Chromaturia (2) | Bladder sphincter atony (2) | Dysuria (1) |
| **Vascular disorders (23)** | Hypotension (16) | Hypertension (5) | Hematoma (1) | Blood pressure inadequately controlled (1) |  |
| **Psychiatric disorders (20)** | Abnormal behavior (9) | Agitation (6) | Disorientation (3) | Apathy (2) | Insomnia (1) |
| **Respiratory, thoracic and mediastinal disorders (10)** | Cough (4) | Catarrh (3) | Oropharyngeal pain (2) | Respiratory disorder (1) |  |
| **Reproductive system and breast disorders (6)** | Pelvic pain (2) | Oedema genital (1) | Scrotal oedema (1) | Testicular pain (1) | Testicular swelling (1) |
| **Ear and labyrinth disorders (6)** | Vertigo (4) | Ear pain (2) |  |  |  |
| **Infections and infestations (5)** | Cellulitis (1) | Conjunctivitis (1) | Pyuria (1) | Sepsis (1) | Necrotizing soft tissue infection (1) |
| **Blood and lymphatic system disorders (2)** | Jaundice acholuric (1) | Lymphadenitis (1) |  |  |  |
| **Immune system disorders (2)** | Anaphylactic shock (1) | Hypersensitivity (1) |  |  |  |
| **Cardiac disorders (1)** | Palpitations (1) |  |  |  |  |
| **Hepatobiliary disorders (1)** | Jaundice (1) |  |  |  |  |
| **Investigations (1)** | Pulse abnormal (1) |  |  |  |  |

**S1C Table. Most frequently reported serious ADR associated with ivermectin for each System Organ Class (SOC) in RoW.** If several sADRs belonging to the same SOC are reported in a single patient (ICSR form), the SOC is counted only once in the total.

| **SOC (number of serious ADR reports)** | **Most frequently reported serious ADR** | | | | |
| --- | --- | --- | --- | --- | --- |
|  | 1^st^ reported (n) | 2^nd^ reported (n) | 3^rd^ reported (n) | 4^th^ reported (n) | 5^th^ reported (n) |
| **General disorders and administration site conditions (176)** | Drug ineffective (38) | Pyrexia (21) | Asthenia (17) | Drug interaction (12) | Fatigue (12) |
| **Skin and subcutaneous tissue disorders (131)** | Pruritus (29) | Rash (22) | Erythema (15) | Toxic epidermal necrolysis (14) | Rash maculo-papular (13) |
| **Nervous system disorders (97)** | Headache (23) | Dizziness (14) | Convulsion (10) | Loss of consciousness (9) | Sleepiness (8) |
| **Gastrointestinal disorders (78)** | Vomiting (18) | Abdominal pain (15) | Nausea (13) | Diarrhea (9à | Dysphagia (7) |
| **Infections and infestations (77)** | Strongyloidiasis (34) | Pneumonia (11) | Sepsis (6) | Acarodermatitis (5) | Conjunctivitis (4) |
| **Injury, poisoning, procedural complications (67)** | Off label use (15) | Accidental exposure (10) | Incorrect route of administration (6) | Exposure during pregnancy (4) | Product use in unapproved indication (4) |
| **Investigations (56)** | Weight decreased (5) | Hepatic enzyme increased (5) | Platelet count decreased (4) | CRP increased (3) | Heart rate increased (3) |
| **Respiratory, thoracic, mediastinal disorders (52)** | Dyspnea (18) | ARDS (7) | Respiratory failure (5) | Asthma (4) | Cough (3) |
| **Hepatobiliary disorders (47)** | Hepatitis (10) | Hepatocellular injury (9) | Liver disorder (5) | Hepatic failure (4) | Abnormal hepatic function (4) |
| **Psychiatric disorders (42)** | Confusional state (13) | Hallucination (4) | Agitation (3) | Delirium (3) | Depression (3) |
| **Blood and lymphatic system disorders (42)** | Eosinophilia (18) | Lymphadenopathy (5) | Agranulocytosis (4) | Anemia (4) | Thrombocytopenia (4) |
| **Renal and urinal disorders (29)** | Acute kidney injury (7) | Hematuria (3) | Kidney failure (3) | Abnormal kidney function (3) | Anuria (2) |
| **Musculoskeletal, connectives tissues disorders (26)** | Pain in extremity (8) | Arthralgia (7) | Back pain (6) | Myalgia (6) | Musculoskeletal weakness (3) |
| **Cardiac disorders (27)** | Cardiac arrest (5) | Tachycardia (5) | Palpitations (4) | Cardiac failure (4) | Cardio-respiratory arrest (3) |
| **Vascular disorders (24)** | Hypotension (4) | Circulatory collapse (2) | Hematoma (2) | Hypertension (2) | Orthostatic hypotension (2) |
| **Metabolism and nutrition disorders (24)** | Decreased appetite (5) | Hyperkaliemia (4) | Dehydration (3) | Hyponatremia (3) | Hyperglycemia (2) |
| **Eye disorders (21)** | Blindness (3) | Vision blurred (3) | Visual impairment (3) | Conjunctival haemorrhage (2) | Ocular irritation (2) |
| **Immune system disorders (13)** | Hypersensitivity (6) | Anaphylactic shock (3) | Anaphylactic reaction (1) | Drug hypersensitivity (1) | Immune system disorder (1) |
| **Pregnancy, puerperium, perinatal disorders (9)** | Abortion spontaneous (4) | Foetal death (2) | Oligohydramnios (1) | Premature delivery (1) | Stillbirth (1) |
| **Neoplasm benign, malignant, unspecified (8)** | Hodgkin’s disease (3) | Hydatidiform mole (1) | Large B-cell lymphoma (1) | Glioblastoma (1) | T-cell lymphoma (1) |
| **Ear and labyrinth disorders (8)** | Vertigo (5) | Tinnitus (2) | Deafness (1) | Ototoxicity (1) |  |
| **Endocrine disorders (7)** | Hypothyroidism (2) | Adrenal insufficiency (1) | Diabetes insipidus (1) | Adrenocortical insufficiency (1) | Autoimmune thyroiditis (1) |
| **Social circumstances (4)** | Loss of independence (2) | Immobile (1) | Impaired driving ability (1) |  |  |
| **Surgical and medical procedures (4)** | Central catheterization (1) | Limb operation (1) | Spinal operation (1) | Mechanical ventilation (1) | Endotracheal intubation (1) |
| **Reproductive system and breast disorders (3)** | Menstruation irregular (1) | Scrotal oedema (1) | Penile swelling (1) | Scrotum swelling (1) |  |
| **Product issues (3)** | Product taste abnormal (1) | Wrong label (1) | Product substitution issue (1) | Product availability issue (1) |  |
| **Congenital, familial and genetic disorders (1)** | Congenital anomaly (1) |  |  |  |  |
