# Supplementary material for: Serious adverse reactions associated with ivermectin: A systematic pharmacovigilance study in sub-Saharan Africa and in the rest of the World
Source: PLoS Negl Trop Dis. 2021 Apr 20;15(4):e0009354. doi: 10.1371/journal.pntd.0009354 (PMC8087035; doi:10.1371/journal.pntd.0009354)
Supplement: S2 Table — (DOCX) [file pntd.0009354.s002.docx]

**S2 Table. Disproportionality analysis of serious adverse reactions associated with ivermectin compared to other antinematodal drugs**

| **Severe adverse drug reaction** | **Drugs** | **Cases** | **Non-cases** | **Crude ROR** (95% CI) | **Adjusted ROR** ^a^ (95% CI) | **Adjusted ROR** ^b^ **in Sub-Saharan Africa** (95% CI) | **Adjusted ROR** ^b^ **in RoW** (95% CI) |
| --- | --- | --- | --- | --- | --- | --- | --- |
| Headache | **Ivermectin** | 71 | 502 | 1.64 ^***^ (1.20–2.25) | 1.45 ^**^ (1.00–2.11) | 1.31 (0.78–2.20) | 1.82 ^**^ (1.01–3.28) |
|  | Antinematodal drugs | 109 | 1265 | Ref. | Ref. | Ref. | Ref. |
| Encephalopathy | **Ivermectin** | 55 | 518 | 9.01 ^***^ (5.12–15.9) | 7.60 ^***^ (4.13–14.0) | 31.6 ^***^ (7.37–135.3) | 4.70 ^***^ (2.25–9.81) |
|  | Antinematodal drugs | 16 | 1358 | Ref. | Ref. | Ref. | Ref. |
| Confusional disorders | **Ivermectin** | 22 | 551 | 3.39 ^***^ (1.77–6.50) | 3.27 ^***^ (1.60–6.69) | 4.82 ^*^ (0.96–24.2) | 2.81 ^***^ (1.28–6.18) |
|  | Antinematodal drugs | 16 | 1358 | Ref. | Ref. | Ref. | Ref. |
| Seizure | **Ivermectin** | 11 | 562 | 0.59 (0.30–1.15) | 0.88 (0.43–1.82) | 0.13 (0.02–1.12) | 1.13 (0.52–2.42) |
|  | Antinematodal drugs | 44 | 1330 | Ref. | Ref. | Ref. | Ref. |
| DRESS ^*^ | **Ivermectin** | 9 | 564 | 10.9 ^***^ (2.36–50.8) | N/A | N/A | N/A |
|  | Antinematodal drugs | 2 | 1372 | Ref. |  |  |  |
| Toxidermia | **Ivermectin** | 25 | 548 | 4.13 ^***^ (2.16–7.90) | 5.41 ^***^ (2.58–11.3) | 0.41 (0.04–4.09) | 4.87^***^ (2.17–10.9) |
|  | Antinematodal drugs | 15 | 1359 | Ref. | Ref. | Ref. | Ref. |
| Psychotic  disorders | **Ivermectin** | 10 | 563 | 1.51 (0.68–3.34) | 1.48 (0.63–3.48) | N/A | 1.51 (0.62–3.71) |
|  | Antinematodal drugs | 16 | 1358 | Ref. | Ref. |  | Ref. |
| Suicidal behavior | **Ivermectin** | 4 | 569 | 0.64 (0.21–1.93) | 0.61 ^*^ (0.20–1.85) | N/A | N/A |
|  | Antinematodal drugs | 15 | 1359 | Ref. | Ref. |  |  |
| SARS ^**^ | **Ivermectin** | 4 | 569 | 10.2 ^**^ (1.13–91.8) | N/A | N/A | N/A |
|  | Antinematodal drugs | 1 | 1373 | Ref. |  |  |  |
| Renal disorders | **Ivermectin** | 17 | 556 | 1.88 ^*^ (0.99–3.56) | 1.37 (0.69–2.69) | N/A | N/A |
|  | Antinematodal drugs | 22 | 1352 | Ref. | Ref. |  |  |
| Hepatic disorders | **Ivermectin** | 50 | 523 | 0.78 (0.56–1.10) | 0.71 (0.50–1.02) | 1.35 (0.10–25.1) | 0.70 (0.49–1.02) |
|  | Antinematodal drugs | 149 | 1225 | Ref. | Ref. | Ref. | Ref. |
| Cardiac failure | **Ivermectin** | 6 | 567 | 2.79 ^*^ (0.83–9.38) | N/A | N/A | N/A |
|  | Antinematodal drugs | 1 | 1373 | Ref. |  |  |  |
| Rhythm disorders | **Ivermectin** | 7 | 566 | 3.39 ^*^ (1.07–10.7) | 2.79 ^*^ (0.83–9.38) | N/A | 3.45 ^**^ (1.02–11.7) |
|  | Antinematodal drugs | 5 | 1369 | Ref. | Ref. |  | Ref. |
| Mazzotti’s reaction | **Ivermectin** | 36 | 537 | 3.62 ^***^ (2.15–6.08) | 2.61 ^***^ (1.41–4.85) | 2.17 ^***^ (1.22–3.88) | 16.5 ^***^ (1.98–137.4) |
|  | Antinematodal drugs | 25 | 1349 | Ref. | Ref. | Ref. | Ref. |

^a^ Adjusted for origin (Sub-Saharan Africa or RoW), gender, age and period of notification; ^b^ Adjusted for gender, age and period of notification; ^*^  Drug reaction with eosinophilia and systemic symptoms; ^**^ Severe Acute Respiratory Syndrome.
