# Supplementary material for: Serious adverse reactions associated with ivermectin: A systematic pharmacovigilance study in sub-Saharan Africa and in the rest of the World
Source: PLoS Negl Trop Dis. 2021 Apr 20;15(4):e0009354. doi: 10.1371/journal.pntd.0009354 (PMC8087035; doi:10.1371/journal.pntd.0009354)
Supplement: S3 Table — (DOC) [file pntd.0009354.s003.doc]

STROBE Statement—Checklist of items that should be included in reports of ***case-control studies***

|  | Item No | Recommendation | Page No |
| --- | --- | --- | --- |
| **Title and abstract** | 1 | (*a*) Indicate the study’s design with a commonly used term in the title or the abstract | Page 1  “systematic pharmacovigilance study” |
|  |  | (*b*) Provide in the abstract an informative and balanced summary of what was done and what was found | Page 2  Abstract |
| Introduction | | | |
| Background/rationale | 2 | Explain the scientific background and rationale for the investigation being reported | Page 5: To our knowledge, these studies are the only two that have used a pharmacovigilance database to evaluate the occurrence of post-ivermectin ADRs and they have focused exclusively on neurological events.  Our systematic search of the literature for non-neurological adverse events found 10 cases where ivermectin was associated with cutaneous reactions [11–15], nephropathy [16], psychiatric disorders [17,18], hepatic disorders [19,20] and multiorgan dysfunction syndrome [21]. Clinical trials and observational studies have reported common adverse events such as headache, pruritus, muscle pain, cough, dyspnea, nausea, vomiting, diarrhea, blurred vision, postural hypotension and confusion and more anecdotal effects such as serious skin reactions and edematous swelling [22–24]. |
| Objectives | 3 | State specific objectives, including any prespecified hypotheses | Page 6 In the present study, we searched VigiBase® for all the suspected sADRs (not only the neurological ones) reported after ivermectin treatment and after treatment with other antinematodal drugs and conducted disproportionality analyses considering the geographical origin of the reported cases. More specifically, the aims of this study were to identify (i) possible non-neurological pharmacovigilance signals (increased reporting of serious suspected adverse reactions after treatment with ivermectin compared to treatment with other antinematodal drugs), and (ii) possible neurological signals related to indications other than onchocerciasis. |
| Methods | | | |
| Study design | 4 | Present key elements of study design early in the paper | Page 7 “We performed disproportionality analyses using the case/non-case method which allows to identify disproportionate reporting, *i.e.* a higher than expected number of adverse reaction reports compared to other reactions recorded in the database by calculating Reporting Odds Ratios (ROR). ROR compares the odds of exposure to ivermectin between cases and non-cases [30,31].  “ |
| Setting | 5 | Describe the setting, locations, and relevant dates, including periods of recruitment, exposure, follow-up, and data collection | Page 7 “All reports of suspected sADRs associated with antinematodal drugs (Anatomical Therapeutic Chemical [ATC] code P02C) from December 2003 (first ever report of ivermectin-associated suspected sADR recorded) up to July 15, 2020 were extracted. Antinematodal drugs included ivermectin, benzimidazole drugs (mebendazole, tiabendazole, albendazole, ciclobendazole, flubendazole, fenbendazole), levamisole, pyrantel, piperazine, diethylcarbamazine, and pyrvinium. Prior to analysis, suspected duplicate reports identified by an automated screening were excluded [28]. When ivermectin had been administered in combination with a benzimidazole or another antinematodal drug, the report was excluded from the analysis. Suspected sADRs were classified following the MedDRA [29], grouped at the System Organ Class (SOC) level and at the individual preferred term (PT) level. |
| Participants | 6 | (*a*) Give the eligibility criteria, and the sources and methods of case ascertainment and control selection. Give the rationale for the choice of cases and controls | Page 7-8 “Cases were defined as reports of each suspected sADR of interest identified by a MedDRA PT for severe headache, encephalopathies, confusional disorders, seizures, toxidermias (drug reaction with eosinophilia and systemic symptoms, Stevens-Johnson syndrome, toxic epidermal necrolysis and acute generalized exanthematous pustulosis), psychiatric disorders, suicidal behavior, severe acute respiratory syndrome (SARS), renal disorders, hepatic disorders, cardiac failure, rhythm disorders and Mazzotti reaction. For specific syndromes of interest, we mapped the PTs for the most common symptoms to one variable and used that in the analyses instead of the individual PTs (Table 1).  Non-cases were defined as reports of any other suspected sADR occurring after administration of the same drug.  “  Page 9 “Exposure to ivermectin was identified in the ICSR by the use of ivermectin (ATC code P02CF01) preceding the onset of the serious adverse reaction. Topical formulations were excluded from the analyses.  “ |
|  |  | (*b*) For matched studies, give matching criteria and the number of controls per case | N/A |
| Variables | 7 | Clearly define all outcomes, exposures, predictors, potential confounders, and effect modifiers. Give diagnostic criteria, if applicable | Page 9 “”To explore a potential effect modification by origin, we performed secondary analyses with stratification according to the origin of the notification (SSA and RoW).  Sensitivity analyses were performed using all antinematodal drugs (including benzimidazoles) as the control group. |
| Data sources/ measurement | 8* | For each variable of interest, give sources of data and details of methods of assessment (measurement). Describe comparability of assessment methods if there is more than one group | Page 6 “Data were extracted from the WHO Global Individual Case Safety Report (ICSR) database, VigiBase® [25] which includes more than 20 million cases of suspected ADRs reported by national pharmacovigilance centers in more than 130 countries participating in the WHO Program for International Drug Monitoring [26]” |
| Bias | 9 | Describe any efforts to address potential sources of bias | Page 9 “Sensitivity analyses were performed using all antinematodal drugs (including benzimidazoles) as the control group.  “ |
| Study size | 10 | Explain how the study size was arrived at | N/A |
| Quantitative variables | 11 | Explain how quantitative variables were handled in the analyses. If applicable, describe which groupings were chosen and why | N/A |
| Statistical methods | 12 | (*a*) Describe all statistical methods, including those used to control for confounding | Page 9 “Descriptive statistics were used to summarize the basic characteristics according to the origin of the ICSR: sub-Saharan Africa (SSA) or the rest of the world (RoW).  Among all suspected sADR reports associated with antinematodal drugs, our primary analyses consisted in calculating the ROR of each suspected sADR of interest (and corresponding 95% confidence interval [95% CI]) for ivermectin compared to benzimidazole drugs using logistic regression models adjusted for age (categorized as less than 18, between 18-65, more than 65 years old, or unknown age), date of the ICSR (2004-2012, 2013-2017 and 2018-2020) and origin of the notification (SSA or RoW) which can be used as a proxy for onchocerciasis or LF being the indication for ivermectin use since >99% of subjects with onchocerciasis live in SSA, and ivermectin is usually not used for LF control/elimination outside SSA. To explore a potential effect modification by origin, we performed secondary analyses with stratification according to the origin of the notification (SSA and RoW).  Sensitivity analyses were performed using all antinematodal drugs (including benzimidazoles) as the control group. “ |
|  |  | (*b*) Describe any methods used to examine subgroups and interactions | Page 9 “To explore a potential effect modification by origin, we performed secondary analyses with stratification according to the origin of the notification (SSA and RoW).  “ |
|  |  | (*c*) Explain how missing data were addressed | Page 9 “For all analyses, the P-value will be represented by asterisks: ***: p < 0.01; **: p < 0.05; *: p < 0.10. For all analyses, "N/A" means that the category is not available or non-applicable” |
|  |  | (*d*) If applicable, explain how matching of cases and controls was addressed | N/A |
|  |  | (*e*) Describe any sensitivity analyses | Page 9 “Sensitivity analyses were performed using all antinematodal drugs (including benzimidazoles) as control group” |
| Results | | | |
| Participants | 13* | (a) Report numbers of individuals at each stage of study—eg numbers potentially eligible, examined for eligibility, confirmed eligible, included in the study, completing follow-up, and analysed | Page 10 “After elimination of duplicates, 2041 suspected sADRs occurring after administration of antinematodal agents were reported between December 2003 and July 2020, of which 209 (10.2%) resulted in death. A total of 667 suspected sADRs were reported after ivermectin administration: 208 cases in SSA and 459 in the RoW” |
|  |  | (b) Give reasons for non-participation at each stage | N/A |
|  |  | (c) Consider use of a flow diagram | N/A |
| Descriptive data | 14* | (a) Give characteristics of study participants (eg demographic, clinical, social) and information on exposures and potential confounders | Page 11 “Most cases concerned people aged 18-44 years old (43.7%) and were reported by healthcare professionals (90.0%). Mean age (44.7 ± 22.9 years for all cases) was significantly lower for SSA (32.3 ± 14.6 years) than for RoW cases (51.1 ± 23.8 years). Sex distribution was also significantly different between SSA cases (female:male ratio 1:1.96) and RoW cases (1:1). Stromectol®, the most frequently brand name reported in the RoW (62.0%), was not reported at all in SSA. Suspected sADRs were more frequently fatal in the RoW (67 deaths; 14.6%) than in SSA (9 deaths; 4.3%). Onchocerciasis was the most frequently reported indication for ivermectin use and this was particularly the case in SSA. Scabies was the second most frequently reported indication for ivermectin use, all cases being from the RoW (96; 28.0%).” |
|  |  | (b) Indicate number of participants with missing data for each variable of interest | N/A |
| Outcome data | 15* | Report numbers in each exposure category, or summary measures of exposure | Page 17 Table 6 |

| Main results | | 16 | (*a*) Give unadjusted estimates and, if applicable, confounder-adjusted estimates and their precision (eg, 95% confidence interval). Make clear which confounders were adjusted for and why they were included | Page 17 Table 6 |
| --- | --- | --- | --- | --- |
|  |  |  | (*b*) Report category boundaries when continuous variables were categorized | N/A |
|  |  |  | (*c*) If relevant, consider translating estimates of relative risk into absolute risk for a meaningful time period | N/A |
| Other analyses | 17 | Report other analyses done—eg analyses of subgroups and interactions, and sensitivity analyses | | Page 17 Table 6 |
| Discussion | | | | |
| Key results | 18 | Summarise key results with reference to study objectives | | Page 18 “Some strong significant disproportionality signals were found, showing more frequent reporting of encephalopathies after ivermectin than after benzimidazoles, both in SSA countries and in the RoW. Disproportionality signals were also identified for serious toxidermias, serious confusional disorders and serious Mazzotti reactions with ivermectin when compared with benzimidazole drugs or all non-ivermectin antinematodal drugs. A less consistent signal was found for cardiac failures and further studies are needed to confirm this result. “ |
| Limitations | 19 | Discuss limitations of the study, taking into account sources of potential bias or imprecision. Discuss both direction and magnitude of any potential bias | | Page 22-23 “Limitations of this study include the concern about under-reporting of suspected ADRs and differences in the under-reporting between different countries as well as the lack of information on the number of drug administrations, which is a major disadvantage inherent in studies using pharmacovigilance databases [33,34]. Although under-reporting may be less important since we focused on serious ADRs (which are more likely to be reported) [35], our analyses cannot measure the real risk of ADR but only the differences in reported events. Indeed, subjects in the control group (non-cases) are not healthy controls but patients with other various reported ADRs and pharmacovigilance data do not consider the total amount of patients exposed to the drug. Nevertheless, there is no apparent reason that, in a specific region, ADRs would be more or less reported with ivermectin than those occurring after treatment with benzimidazole drugs or other antinematodal drugs. By analyzing real-life surveillance data, disproportionality analyses have demonstrated their usefulness for detecting drug risks [36,37]. Anyway, these results should be taken with caution because of potential missing information. Pharmacovigilance systems are not yet well established in SSA countries. In 2017, only 30% of these countries had specific procedures for the monitoring of ADRs and only 28% had a platform for coordinating pharmacovigilance activities at the national level [38]. Cases of serious adverse events occurring during the ivermectin MDA organized by the onchocerciasis and LF control programs have to be reported by the countries to the Mectizan Donation Program, but the extent to which all relevant observations are recorded in the rural areas where onchocerciasis and LF are endemic and then passed on to the central level is unknown as is the extent to which they are reported into the WHO VigiBase. For example, we found no cases from Cameroon even though ivermectin MDA programs have been ongoing there for 30 years, and many cases are known to have occurred since the early 1990s [39]. In addition, the first case of a post-ivermectin ADR was reported in VigiBase® in December 2003 while the first reported death after ivermectin was reported by the WHO Drug Information in 1991 [40]. We consider it likely that availability of complete data from SSA would show more cases associated with ivermectin use and would increase the strength of the safety signals we identified. “ |
| Interpretation | 20 | Give a cautious overall interpretation of results considering objectives, limitations, multiplicity of analyses, results from similar studies, and other relevant evidence | | Page 18-19 “Some of these results were expected, given the different mechanisms of action of ivermectin and benzimidazoles on the various targeted parasites. Ivermectin exerts a strong microfilaricidal effect on filariae, leading to a destruction of mf within one week after treatment. In subjects infected with *Onchocerca volvulus*, the destruction of mf in the skin is associated with inflammatory processes leading to the so-called Mazzotti reaction. In those infected with *L. loa*, the drug probably induces a paralysis of the *L. loa* mf, which are then drained passively in the blood circulation. If the MFD is high, the process can lead to an embolization of mf in the brain capillaries, to inflammatory reactions at the cerebral level, and to an encephalopathy. In contrast, benzimidazoles have little short-term effect on the mf of any filarial species, and thus do not induce Mazzotti reactions, but impair the production of new mf by the adult female worms.  “ |
| Generalisability | 21 | Discuss the generalisability (external validity) of the study results | | Page 23 “In addition, a notoriety bias (selection bias in which a case has a greater chance of being reported if the drug is known to cause, thought to cause, or likely to cause the event of interest [41]) could be considered for reports of encephalopathy in SSA given that the first cases of encephalopathies involving ivermectin led to complications in the early MDA campaigns for elimination of onchocerciasis. However it is unlikely that such bias exist for two reasons (i) in SSA countries, ivermectin is distributed as part of mass treatment organized by the Ministries of Health, and those of *L. loa*-endemic countries might be less inclined to report post-ivermectin sADRs because the cases are not regarded as exceptional and (ii) we also found a strong disproportionality signal in the RoW which is not being affected by this bias .  “ |
| Other information | | | | |
| Funding | 22 | Give the source of funding and the role of the funders for the present study and, if applicable, for the original study on which the present article is based | | Page 25 “The author(s) received no specific funding for this work  “ |

*Give information separately for cases and controls.

**Note:** An Explanation and Elaboration article discusses each checklist item and gives methodological background and published examples of transparent reporting. The STROBE checklist is best used in conjunction with this article (freely available on the Web sites of PLoS Medicine at http://www.plosmedicine.org/, Annals of Internal Medicine at http://www.annals.org/, and Epidemiology at http://www.epidem.com/). Information on the STROBE Initiative is available at http://www.strobe-statement.org.
