# Supplementary material for: Serious adverse reactions associated with ivermectin: A systematic pharmacovigilance study in sub-Saharan Africa and in the rest of the World
Source: PLoS Negl Trop Dis. 2021 Apr 20;15(4):e0009354. doi: 10.1371/journal.pntd.0009354 (PMC8087035; doi:10.1371/journal.pntd.0009354)
Supplement: Attachment — Submitted filename: Reviewer attachment_PLOS.docx [file pntd.0009354.s004.docx]

**Reviewer attachment:**

The study will benefit so many people within the healthcare field, as it discusses the adverse events of ivermectin that have been reported during an 18 years period. The disproportionality method used is novel for this drug and its limitations have been clarified and discussed by the authors as well as its strengths. The science is sound and the language is clear. This study also highlights the importance of proper pharmacovigilance procedures needed in many different parts of the world where the sub-Saharan Africa is provided as an example.

I have a few points to be considered by the authors. I have included them in their respective sections.

**Editorial and Data Presentation Modifications**

1- The title can be rewritten to reflect the study design where ivermectin pharmacovigilance study was compared between sub-Saharan Africa and the rest of the world and not it was not limited to a comparison between "sub-Saharan Africa and non-sub-Saharan African regions"

2- key words: "antinematodal drugs" can be added to the keywords for better search results.

3- Page 4 line 79-83. The paragraph needs rewriting for clarification. This paragraph also needs a reference and adding the route of administration for MDA strategy

4- Page 5 line 102: there is no need for the sentence "see below".

**In the methodology Part:**

Methodology can be improved by:

1- clarifying the purpose and statistical tools used for the sensitivity test performed (page 9)

2- on page 9 line 176: Exposure definition would be clearer if it were defined by the included route of administration in addition to the excluded route of administration.

3- Page 9 lines 187 - 189: the categories for ages and for date of the ICSR are not really essential as they are clear enough in the tables. A suggested paraphrasing would be:

"---drugs using logistic regression models adjusted for age groups, date of the ICSR publication, and origin of the notification (SSA or RoW). The latter can additionally be used as a proxy for the ivermectin indication and for the compared regions

**In the results part:**

Here are some suggested points for the results:

1- Page 13 line 253 "i" in ICSRs should be capitalized.

2-When discussing the disproportionality results, the authors can clarify the results better if they order the results in descending order i.e. starting from those that are more significant to the least significant. This order can be applied to the table as well.

3- Page 15 line 288 "serious seizures were not more ..... benzimidazole drugs". Can the authors clarify this sentence? This also applies to the sentence on page 16 lines 299 -300, and lines 301- 307. Clarifying these sentences will definitely improve the results section.

4- Table 6 and table S2: can the authors clarify the "1[Reference]"

5- Table S2 the title is "Disproportionality analysis of serious adverse reactions associated with ivermectin compared to other antinematodal drugs" but the drugs included for comparison are benzimidazoles.
