# Supplementary material for: Serious adverse reactions associated with ivermectin: A systematic pharmacovigilance study in sub-Saharan Africa and in the rest of the World
Source: PLoS Negl Trop Dis. 2021 Apr 20;15(4):e0009354. doi: 10.1371/journal.pntd.0009354 (PMC8087035; doi:10.1371/journal.pntd.0009354)
Supplement: Attachment — Submitted filename: Cover_Letter_PV_Review.docx [file pntd.0009354.s005.docx]

Editor-in-chief

PLoS Neglected Tropical Diseases

March the 10^th^, 2021

Dear Editor-in-chief,

We hereby submit the revised version of our manuscript initially entitled “Serious adverse reactions associated with ivermectin: a systematic pharmacovigilance study in sub-Saharan Africa and non-sub-Saharan African regions” (PNTD-D-21-00099), whose title was revised following a reviewer’s comment. We thank the three reviewers for their positive comments and appreciation of the paper.

We addressed each of the editor’s comments with detailed responses. We hope our manuscript will now meet the standards for publication.

Best regards,

Jérémy Campillo

I. **Reviewer 1:**

The study will benefit so many people within the healthcare field, as it discusses the adverse events of ivermectin that have been reported during an 18 years period. The disproportionality method used is novel for this drug and its limitations have been clarified and discussed by the authors as well as its strengths. The science is sound and the language is clear. This study also highlights the importance of proper pharmacovigilance procedures needed in many different parts of the world where the sub-Saharan Africa is provided as an example.

I have a few points to be considered by the authors. I have included them in their respective sections.

Editorial and Data Presentation Modifications

1- The title can be rewritten to reflect the study design where ivermectin pharmacovigilance study was compared between sub-Saharan Africa and the rest of the world and not it was not limited to a comparison between "sub-Saharan Africa and non-sub-Saharan African regions"

Response: We agree with this comment, we modified the title for: “Serious adverse reactions associated with ivermectin: a systematic pharmacovigilance study in sub-Saharan Africa and the rest of the World”

2- key words: "antinematodal drugs" can be added to the keywords for better search results.

Response: We thank the reviewer for this comment. We agree and will add this keyword in the resubmission.

3- Page 4 line 79-83. The paragraph needs rewriting for clarification. This paragraph also needs a reference and adding the route of administration for MDA strategy

Response: We have clarified the sentence according to the reviewer's proposals (line 80-86).

4- Page 5 line 102: there is no need for the sentence "see below".

Response: We have removed these words.

In the methodology Part:

Methodology can be improved by:

1- clarifying the purpose and statistical tools used for the sensitivity test performed (page 9)

Response: We clarified the sentence to explain that we used the same method as in the primary analysis for this sensitivity analysis (line 195-196).

2- on page 9 line 176: Exposure definition would be clearer if it were defined by the included route of administration in addition to the excluded route of administration.

Response: We clarified the sentence (line 180-181).

3- Page 9 lines 187 - 189: the categories for ages and for date of the ICSR are not really essential as they are clear enough in the tables. A suggested paraphrasing would be: "---drugs using logistic regression models adjusted for age groups, date of the ICSR publication, and origin of the notification (SSA or RoW). The latter can additionally be used as a proxy for the ivermectin indication and for the compared regions

Response: We revised the sentence according to the reviewer proposition (line 188-192).

In the results part:

Here are some suggested points for the results:

1- Page 13 line 253 "i" in ICSRs should be capitalized.

Response: We capitalized the “i”.

2- When discussing the disproportionality results, the authors can clarify the results better if they order the results in descending order i.e. starting from those that are more significant to the least significant. This order can be applied to the table as well.

Response: We decided to present the disproportionality results ordered by SOC. We feel it more relevant to choose this presentation so that readers can directly read the part concerning the SOC they are more interested on.

3- Page 15 line 288 "serious seizures were not more ..... benzimidazole drugs". Can the authors clarify this sentence? This also applies to the sentence on page 16 lines 299 -300, and lines 301- 307. Clarifying these sentences will definitely improve the results section.

Response: We also presented aRORs for serious adverse drug reactions which are not associated with ivermectin intake.

4- Table 6 and table S2: can the authors clarify the "1[Reference]"

Response: We replaced "1[Reference]" by “Ref.” for clarification.

5- Table S2 the title is "Disproportionality analysis of serious adverse reactions associated with ivermectin compared to other antinematodal drugs" but the drugs included for comparison are benzimidazoles.

Response: We have corrected this error in the S2 table.

II. **Reviewer 2:**
1- Line 259, “table 4” dizziness is duplicated. should be removed

Response: We have corrected this error.

2- No comments regarding ethical approval was mentioned.

Response: This study was conducted using the Vigibase^®^ database, which is a database accessible to any pharmacovigilance service and does not require an ethical application because all data are anonymized.

3- Line 75 “infection with Wuchereria bancrofti” is an off-label use in North America (FDA) and is labeled for use in only in Europe (EMA), the same in line 77 “human sarcoptic scabies”. This is contradicting with line 74 “Europe and North America” which refers to labeled use in both, also contradicting with line 346. So, from line 74 till line 79 should be reframed.

Response: We thank the reviewer for this clarification. We have clarified this part according to the information provided by the reviewer (line 74-80).

4- Abbreviations overuse (probably to reduce word count) which may confuse some readers keeping low clarity of the manuscript.

Response: We thank the reviewer for this comment. We removed some abbreviations but decided to keep those commonly used in our disciplinary field (ICSR, ADR, ROR…). However, we are ready to remove other abbreviations at the request of the editor.

III. **Reviewer 3:**
1- Ethical committee permission?

Response: This study was conducted using the Vigibase^®^ database, which is a database accessible to any pharmacovigilance service and does not require an ethical application because all data are anonymized.
